# Supplementary material for: Two opposing hippocampus to prefrontal cortex pathways for the control of approach and avoidance behaviour
Source: Nat Commun. 2022 Jan 17;13:339. doi: 10.1038/s41467-022-27977-7 (PMC8763938; doi:10.1038/s41467-022-27977-7)
Supplement: Supplementary file 1 — Supplementary information [file 41467_2022_27977_MOESM1_ESM.pdf]

Two opposing hippocampus to prefrontal cortex pathways for the control of approach and avoidance behavior

Candela Sánchez-Bellot[1], Rawan AlSubaie[1#], Karyna Mishchanchuk[1#], Ryan W. S. Wee[1] and Andrew F. MacAskill [1*]

Supplementary Information

SUPPLEMENTARY FIGURES

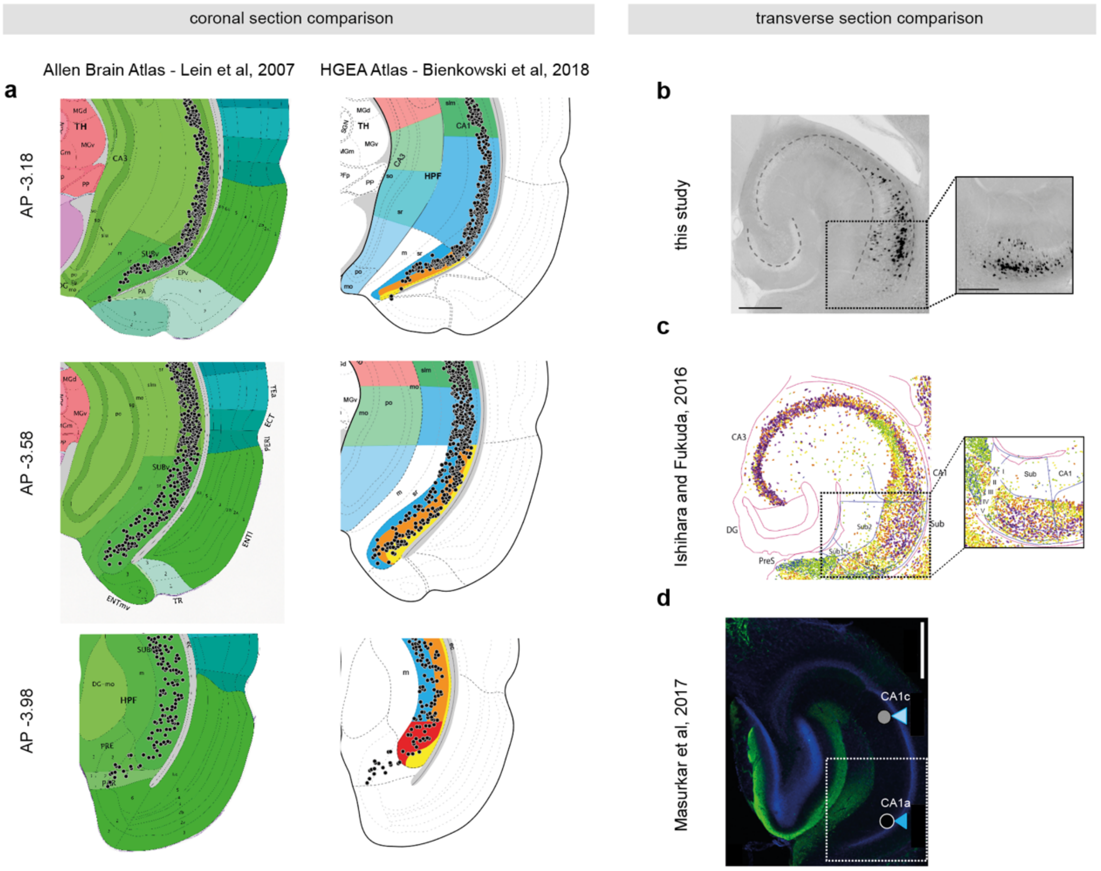

Supp Fig.1 | Distribution of vH PFC-projecting cells compared with published atlases and hippocampal studies

a) *Left,* dots represent segmented cells from the CTXβ labelling experiment using WholeBrain aligned to coronal sections from the Allen Brain Atlas for three different AP planes. Note that within subiculum and more posteriorly, PFC-projecting cells segregate into two layers along the radial (ML here) axis. *Right*, the same cells aligned to coronal sections from the recently published HGEA atlas, showing more detail of layers within subiculum.

b) Distribution of CTXβ labelled vH-PFC cells from this study shown in a transverse section. As the proximal:distal or radial axis of the hippocampal circuit is oriented in the transverse plane, this orientation aids in characterizing the distribution of vH-PFC cells more accurately. Note the segregation of cells into two layers at the extreme superficial and deep poles of the radial axis. Image as in Figure 1, representative of slices from 5 injections. Scale bars = 500 μm.

c) Schematic of hippocampal subregions and layers in a transverse plane of vH at interaural level 1.8 mm (DV ~ -3.9) in the Paxinos Brain Atlas, adapted from Ishihara and Fukuda, 2016. In this study, immunohistochemical investigation is used to characterize the mouse subiculum and its border with CA1. Note that the area we focused on for our study and where the two-layered distribution of vH-PFC is most obvious is denominated subiculum and is composed of 5 layers, as opposed to the 3 layers (layer II, III and IV) present in vH of the HGEA atlas.

d) An example image of a transverse hippocampal slice taken from Masurkar et al. 2017. In this study, authors use hippocampal nomenclature established by Lorente de Nó (1934), dividing CA1 into three equally sized sections, with CA1a being the most distal, bordering with subiculum. The same area as in **b)** and **c)** is boxed for comparison. Following the nomenclature used in this study, vH-PFC cells would be absent from subiculum and occupying CA1c to CA1a. Scale bar = 500μm. Figure adapted from Masurkar et al., 2017.

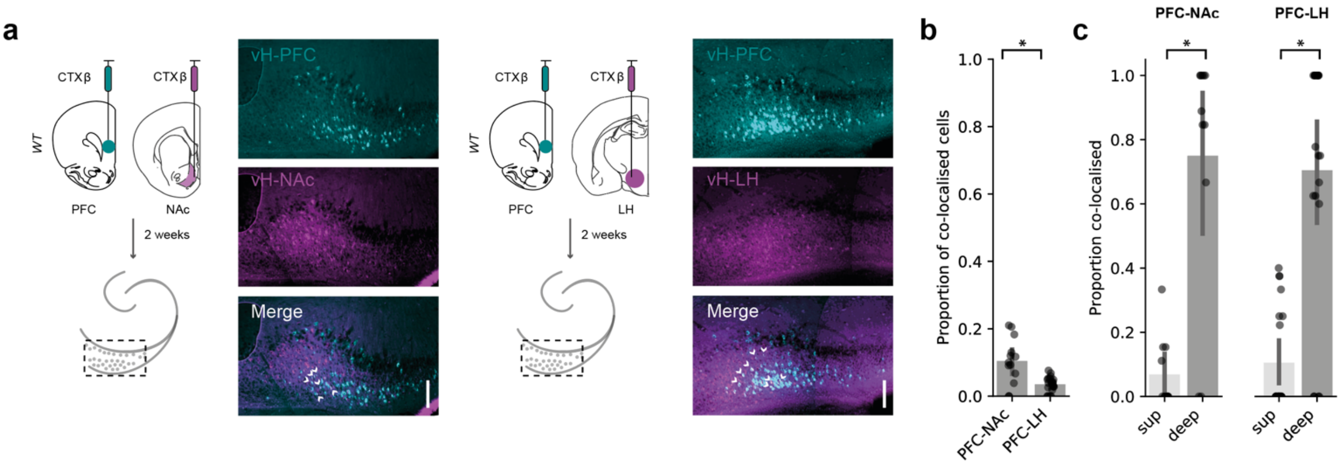

Supp Fig.2 | Collateralization within the vH-PFC population is low and biased towards the deep layer

a) *Left*, Example images of the hippocampal field straightened using the FIJI *Straighten* plug-in with CTXβ labelling tagged with different fluorescent markers for PFC-projecting (vH-PFC, *top*) and NAc-projecting (vH-NAc, *middle*) cells. *Bottom* panel shows both channels merged, and arrowheads point at co-labelled cells. *Right*, the same experiment repeated for PFC- and LH-projecting cells. *Bottom* panel shows the merged image with both channels, and arrowheads point at cells co-labelled with both tracers, projecting to both PFC and LH. Note that the vast majority of co-labelled cells are found in the deep layer of the PFC-projection population. Images representative of 13 (NAc) and 21 (LH) slices quantified below. Scale bar = 100 μm.

b) Quantification of cells with co-localized CTXβ labelling for the PFC-NAc and the PFC-LH experiments. The proportion of co-labelled cells was calculated as a proportion of the total number of PFC-projecting cells in the slice. Note that the overall level of co-localization is very low in the two experiments, although there is slightly more overlap with NAc projections (two-sided Welch's t-test, $t_{(13.6)} = 3.5$, $*p = 0.004$, $n = 13$ (NAc), 21 (LH) slices from 4 (NAc) and 6 (LH) injections).

c) *Left*, quantification of the proportion of co-localized cells for the PFC-NAc experiment (those summarized in **b)**) within each layer of the PFC-projection population. Note that although overall co-localization is very low, those cells that do collateralize are more prevalent in the deep layer (two-sided Welch's paired t-test, $t_{(20)} = 5.6$, $*p = 0.00002$, $n = 11$ slices from 4 injections), with a negligible proportion present in the superficial layer. *Right*, as in the left panel but for PFC-LH collateralization. Note the similar distribution of co-localized cells, which are biased towards the deep layer (two-sided Welch's paired t-test, $t_{(40)} = 6.7$, $*p = 5.3\times10^{-8}$, $n = 21$ slices from 6 injections).

Bar charts show mean (bar) and SEM (error).

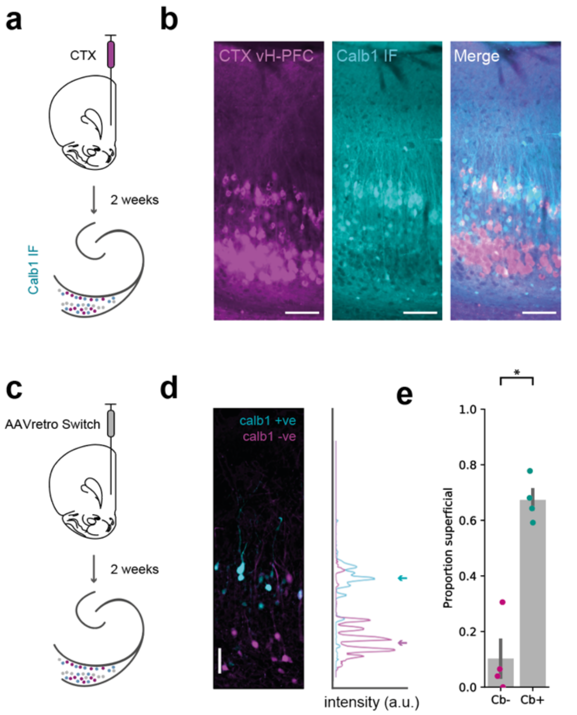

Supp Fig.3 | Characterization of Calbindin1 expression in vH

a) Schematic of CTXβ injection into PFC and retrograde labelling combined with Calbindin 1 immunofluorescence (Calb1 IF) in vH.

b) *Left*, zoom in of the CA1 / subiculum border area of a transverse hippocampal slice with CTXβ labelling vH-PFC cells. *Middle*, Calbindin1 immunofluorescence staining carried out on the same slice. Note how the fluorescence is restricted to the superficial hippocampal layers. *Right*, merge of both channels showing Calb1 staining co-localised with superficial layer vH-PFC cells. Image representative of 3 injections. Scale bar = 75 μm.

c) Schematic of *AAVretro-Switch* injection into the PFC of *Calb1-Cre* mice and subsequent cre-dependent retrograde labelling in hippocampus.

d) *Left,* zoom of retrogradely labelled neurons in the CA1 / subiculum border area of a transverse hippocampal slice, with fluorescence intensity profile for *Calb1*$^{+}$ (*cyan*) and *Calb1*$^{-}$ (*magenta*) neurons on *right*. Arrows highlight the two genetically distinct peaks of fluorescence within the PFC-projection population at the two extremes of the radial axis. Image representative of 4 injections. Scale bar = 50 μm.

e) Quantification of the proportion of Calbindin1-negative (Cb-) and Calbindin1-positive (Cb+) cells in the superficial layer of the vH-PFC projection population. Note that the majority of Cb+ cells are present in the superficial vH-PFC layer (two-sided Welch's t-test, $t_{(6)} = 7.2$, $*p = 0.0004$, $n = 4$ injections), consistent with **b)**, making the expression of Calbindin1 a suitable strategy to differentiate between the two layers of PFC-projecting cells.

Bar charts show mean (bar) and SEM (error).

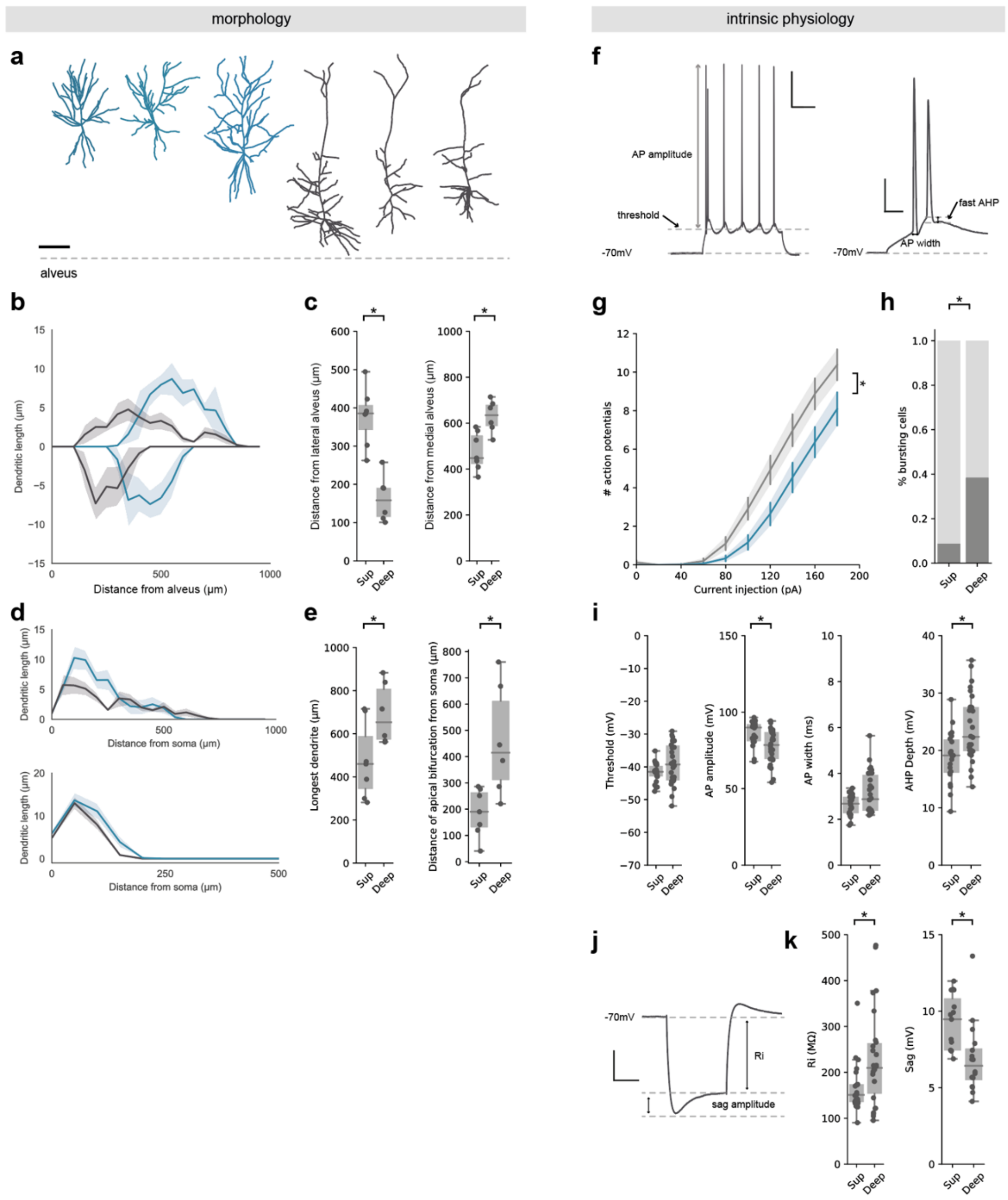

Supp Fig.4 | Characterization of superficial and deep vH-PFC neurons

a) Example reconstructions of superficial (blue) and deep (dark grey) PFC-projecting hippocampal neurons. Scale bar = 100 μm, dotted line represents alveus. Note the complex branched morphology of superficial layer (blue) PFC-projecting cells in contrast to longer and more sparsely branched deep layer (grey) PFC-projecting cells. Images representative of 7 superficial and 6 deep neurons.

b) Sholl analysis showing average dendritic length of apical (top) and basal (bottom) dendrites with increasing distance from the lateral alveus for superficial (blue) and deep (grey) layer vH-PFC neurons. Each population samples distinct range of the radial hippocampal axis.

c) Quantification of distance of the soma of recorded neurons in deep and superficial layers from the lateral alveus (towards cortex – *left*, two-sided Mann Whitney, U = 0, p = 0.0004), and medial alveus (towards dentate gyrus – *right*, two-sided Mann Whitney, U = 39, p = 0.012, n = 7 (sup), 6 (deep) neurons from 6 mice).

d) Sholl analysis of apical (top) and basal (bottom) dendrites with increasing distance from the soma for superficial (blue) and deep (grey) layer vH-PFC neurons. Superficial neurons have more complex dendritic trees.

e) *Left,* quantification of the distance from the farthest dendrite tip to the soma in superficial and deep vH-PFC neurons (two-sided Mann Whitney, U = 35.5, p = 0.045) *Right,* quantification of the distance of the apical bifurcation from the soma in superficial and deep neurons (two-sided Mann Whitney, U = 38.5, p = 0.015, n = 7 (sup), 6 (deep) neurons from 6 mice). Deep layer neurons have longer apical dendrites that branch further from the soma.

f) Example voltage traces from a deep layer PFC-projecting cell in response to current injections (140 pA). Trace on right is a zoom in of the first two events of the first trace. Arrows point at different aspects of the traces analyzed below. Scale bars = 100 ms, 20 mV; 10 ms, 20 mV.

g) Quantification of the number of action potentials elicited by somatic current injection in superficial (blue) and deep (black) neurons (Repeated measures ANOVA with Greenhouse-Geisser correction, Interaction between group and current step: $F_{(1.4,\ 44.5)}$ = 4.3, *p = 0.032, n = 22 (sup) and 26 (deep) from 12 mice).

h) Quantification of the proportion of bursting neurons after positive current injections (Fishers Exact test, Odds ratio = 12.5, *p = 0.009). Note that deep layer cells have higher excitability.

i) Quantification of (*left to right*) action potential (AP) threshold, AP amplitude, AP half-width, and depth of the fast after hyperpolarization (AHP) (two-sided Mann Whitney, AP threshold: U = 219, p = 0.169; AP amplitude: U = 423, *p = 0.005; AP width: U = 207, p = 0.104; AHP depth: U = 143, *p = 0.003, n = 22 (sup) and 26 (deep) from 12 mice).

j) Example voltage trace from a deep layer PFC-projecting cell in response to negative current injections (-160 pA). Arrows point at different aspects of the traces analyzed below. Sacle bar = 200 ms, 10 mV.

k) Quantification of input resistance and sag amplitude after negative current injections (two-sided Mann Whitney, Ri: U = 393, *p = 0.028; sag: U = 39.5, *p = 0.002, n = 22 (sup) and 26 (deep) from 12 mice).

Box plots show median, 75th (box) and 95th (whiskers) percentile. Line plots show mean (line) and SEM (shading and error bars).

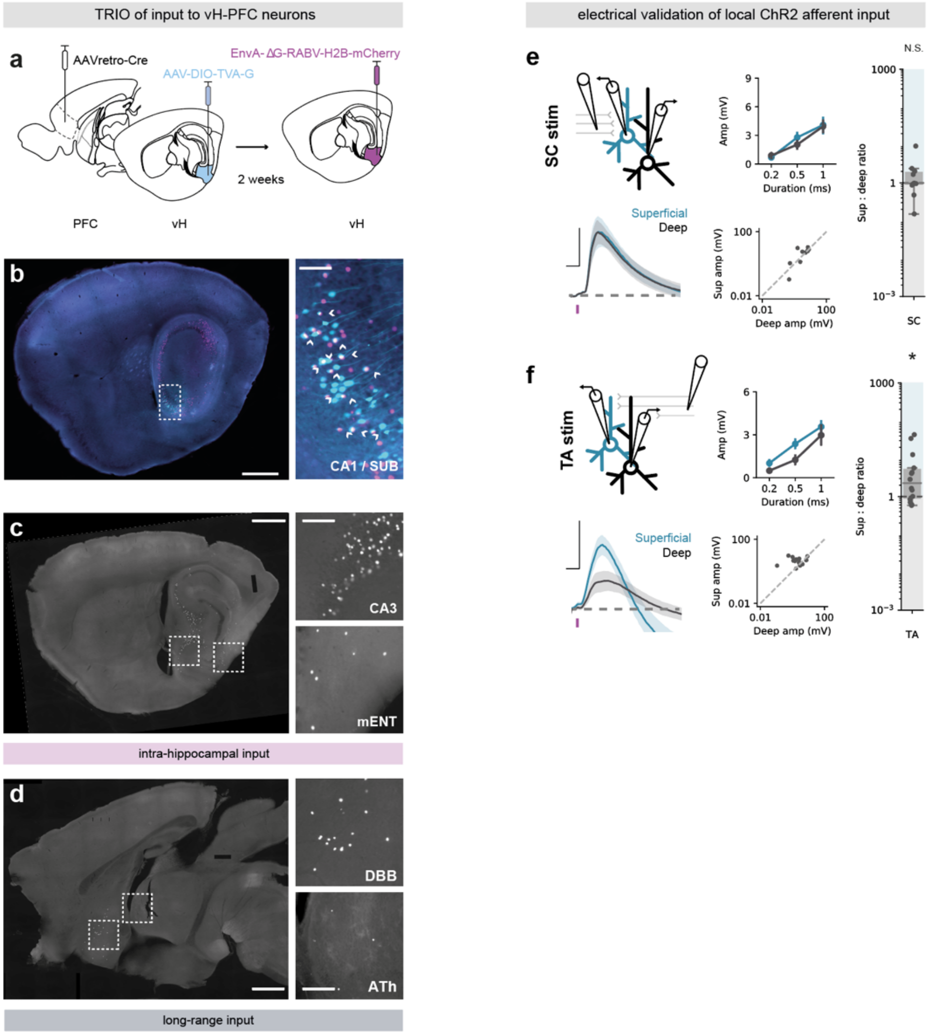

Sup. Fig. 5. | Quantification of TRIO-labelled inputs and electrically stimulated validation of CA3 and ENT input

- **a)** Schematic of TRIO injection strategy. AAVretro-CAG-Cre was injected in PFC, and rabies helper proteins were injected into vH to limit subsequent rabies infection to hippocampal PFC projection neurons. 2 weeks later, EnvA pseudotyped rabies was injected in vH to label presynaptic neurons that synapse onto PFC-projecting hippocampal neurons with nuclear-localized mCherry.
- **b)** *Left,* Injection site in a sagittal section showing TVA and G protein expressing hippocampal neurons (cyan), and rabies labelled neurons (magenta). Scale bar = 1000 μm. *Right,* Zoom in to white dotted box. Co-labelled neurons represent starter neurons (arrows). Image representative of 4 injections. Scale bar = 100 μm.
- **c)** Sagittal section showing insets of rabies-labelled cells in intra-hippocampal input regions CA3 and mENT. Image representative of 4 injections. Scale bar = 1000 μm, 200 μm.
- **d)** Sagittal section showing insets of rabies-labelled cells in extra-hippocampal input regions DBB and ATh. Image representative of 4 injections. Scale bar = 1000 μm, 200 μm
- **e)** *Top left,* Schematic showing experimental setup. Retrobeads were injected into PFC. 2 weeks later Schafer collaterals (SC) were electrically stimulated to mimic ipsilateral CA3 activity, and connectivity was assessed using paired recordings of superficial and deep vH-PFC neurons in acute slices.
  Bottom left, Average stimulation-evoked responses in superficial (blue) and deep (black) layer PFC-projecting hippocampal neurons in response to CA3 input. Scale bar = 10 ms, 2 mV. Purple tick represents the time of stimulus.
  Middle, summary of amplitude of Sup and Deep responses to increasing stimulus intensity (*top*), and amplitudes of individual pairs at 0.5 ms duration (*bottom*).
  Right, summary of the ratio of superficial : deep neuron EPSP. Higher values mean input is biased to superficial neurons, low values towards deep layer neurons. Note log scale. CA3 input is equivalent onto superficial and deep layer neurons (two-sided Wilcoxon paired test, W = 23, p = 0.646, n = 10 pairs of neurons from 3 mice).
- **f)** As in (**e**) but for stimulation of temporoammonic axons to mimic ENT activity. Scale bar = 10 ms, 2 mV. Consistent with light-evoked stimulation in **Figure 3**, ENT input is biased towards activation of superficial layer neurons (two-sided Wilcoxon paired test, W = 19, p = 0.035, n = 14 pairs of neurons from 3 mice).

Box plots show median, 75th (box) and 95th (whiskers) percentile. Line plots show mean (line) and SEM (shading and error bars).

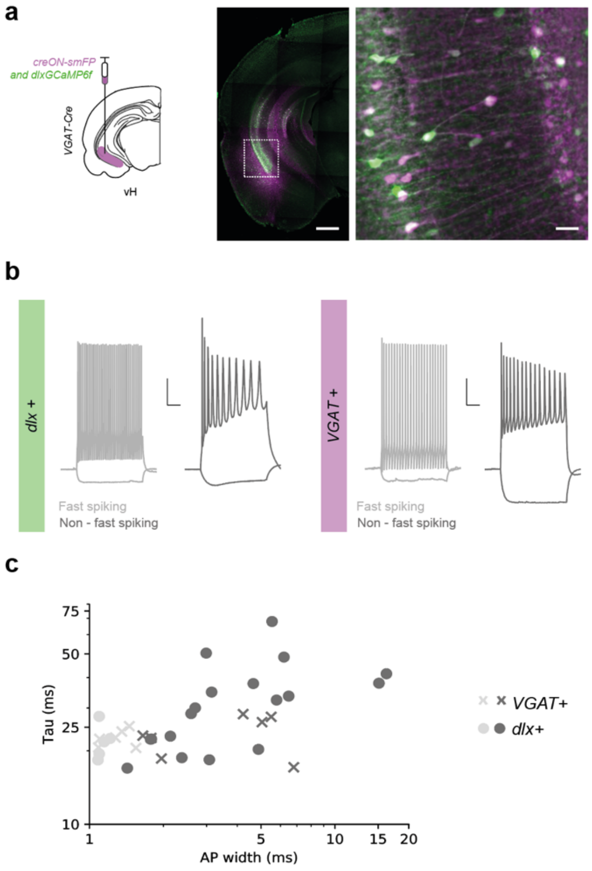

Supp Fig. 6 | Validation of strategy to target inhibitory interneurons in vH

- **a)** *Left*, schematic of *creON-smFP* and *dlx-GCaMP6f* injections into vH of a *VGAT*-cre mouse to label *VGAT*+ cells with smFP and *dlx*+ neurons with GCaMP6f. *Middle*, coronal section showing smFP (magenta) and GCamP6f (green) labelling in vH. *Right*, zoom of boxed region in middle panel, showing overlap of smFP and GCaMP6f labelling. Image representative of 3 injections. Scale bars 500 μm (*right*), 20 μm (*left*).
- **b)** Example recordings in response to positive (+140 pA, upwards traces) and negative (-30 pA, downward traces) current injections for fast spiking and non-fast spiking interneurons recorded from *dlx*+ (*left*) and *VGAT*+ (*right*) cells in vH.
- **c)** Summary showing clustering of recorded interneurons into two groups (fast spiking and non-fast spiking) based on AP width and membrane time constant Tau. Note that the distribution into two groups is similar for *dlx*+ and *VGAT*+ cells.

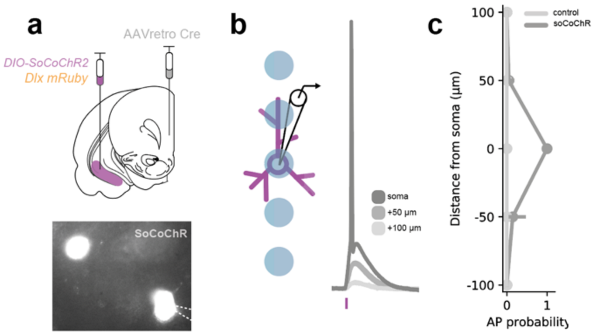

Supp Fig. 7 | Validation of the spatial resolution of somatic ChR2 used for local CRACM experiments

a) *Top,* Strategy to investigate superficial and deep vH neuron connectivity onto local interneurons. Retrogradely transported cre was injected into PFC and cre-dependent somatic ChR2 (DIO-soCoChR2) together with dlx mRuby were injected into vH. *Bottom*, example image of two soCoChR2-positive cells in an acute vH slice. Image representative of all cells recorded. Note recording pipette on bottom cell for characterisation shown in **(b)**.

b) Schematic of experimental setup and example voltage traces showing the effects of light stimulation on the soma, and 50 and 100 *µ*m removed from soCoChR2+ cells. Purple tick represents light stimulus. Note how focused blue light allows activation of neurons expressing soCoChR2 with high spatial resolution.

c) Summary graph showing the probability of eliciting an action potential in soCoChR2-positive cells by shining brief 5 ms pulses of blue light at varying distances from the soma. Controls were taken as activity recorded during the 100 ms prior to stimulation in soCoChR2-positive cells.

Line plots show mean (line) and SEM (error bars).

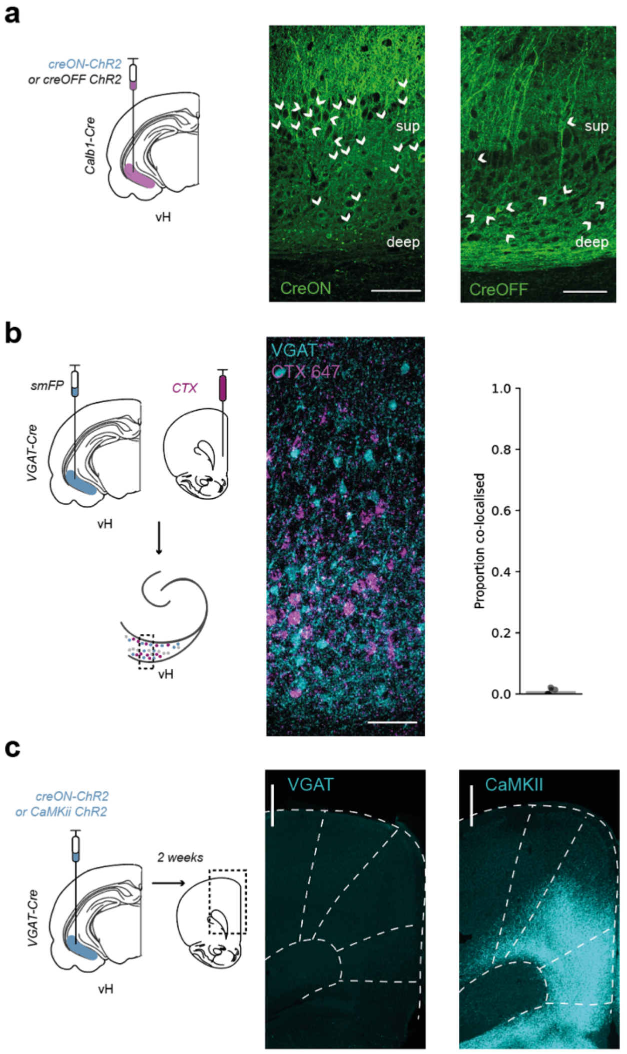

Supp Fig. 8 | Validation of viral strategies used for local and long-range CRACM experiments

a) *Left,* schematic of viral strategy used for long-range CRACM and *in vivo* optogenetics experiments. In Calb1-cre mice, either creON- or creOFF-ChR2 constructs were injected in vH to be expressed in superficial (cre+) and deep (cre-) hippocampal layers, respectively. *Middle* and *right*, example images of creON and creOFF ChR2 in transverse hippocampal slices. Arrowheads point at ChR2+ cells. Note the differential distribution of creON ChR2 (preferentially in the superficial layer) and creOFF (preferentially in the deep layer). Images representative of 3 injections. Scale bars = 100μm.

b) *Left,* schematic of cre-dependent smFP tracer into vH and CTXβ injection into PFC of a VGAT-Cre mouse and subsequent smFP and retrograde CTXβ labelling in vH. *Middle*, example image of cre-dependent smFP (and thus VGAT+) and CTXβ labeling in a transverse hippocampal slice. Note the absence of co-localization of the two tracers. Images representative of 2 injections. Scale bar = 100 μm. *Right*, quantification of co-localized (co-labelled) cells as a proportion of all CTX+ cells.

c) *Left,* Schematic of strategy to label axon terminals from inhibitory and excitatory vH cells with an injection of creON or CaMKii-ChR2 in vH in a VGAT-Cre mouse respectively, for subsequent visualization in PFC. CreON-ChR2 will label terminals from VGAT+ cells while CaMKii-ChR2 will label terminals from excitatory cells. *Middle* and *right*, example images of fluorescence in PFC following these injections showing that no VGAT+ vH axon terminals are present while CaMKii+ vH axon terminals strongly innervate PFC. Images representative of 3 injections. This suggests that calbindin + inhibitory neurons do not send long-range inhibitory input to PFC and that calbindin1+ input studied here is excitatory.

Line plot show mean (line) and SEM (error bars).

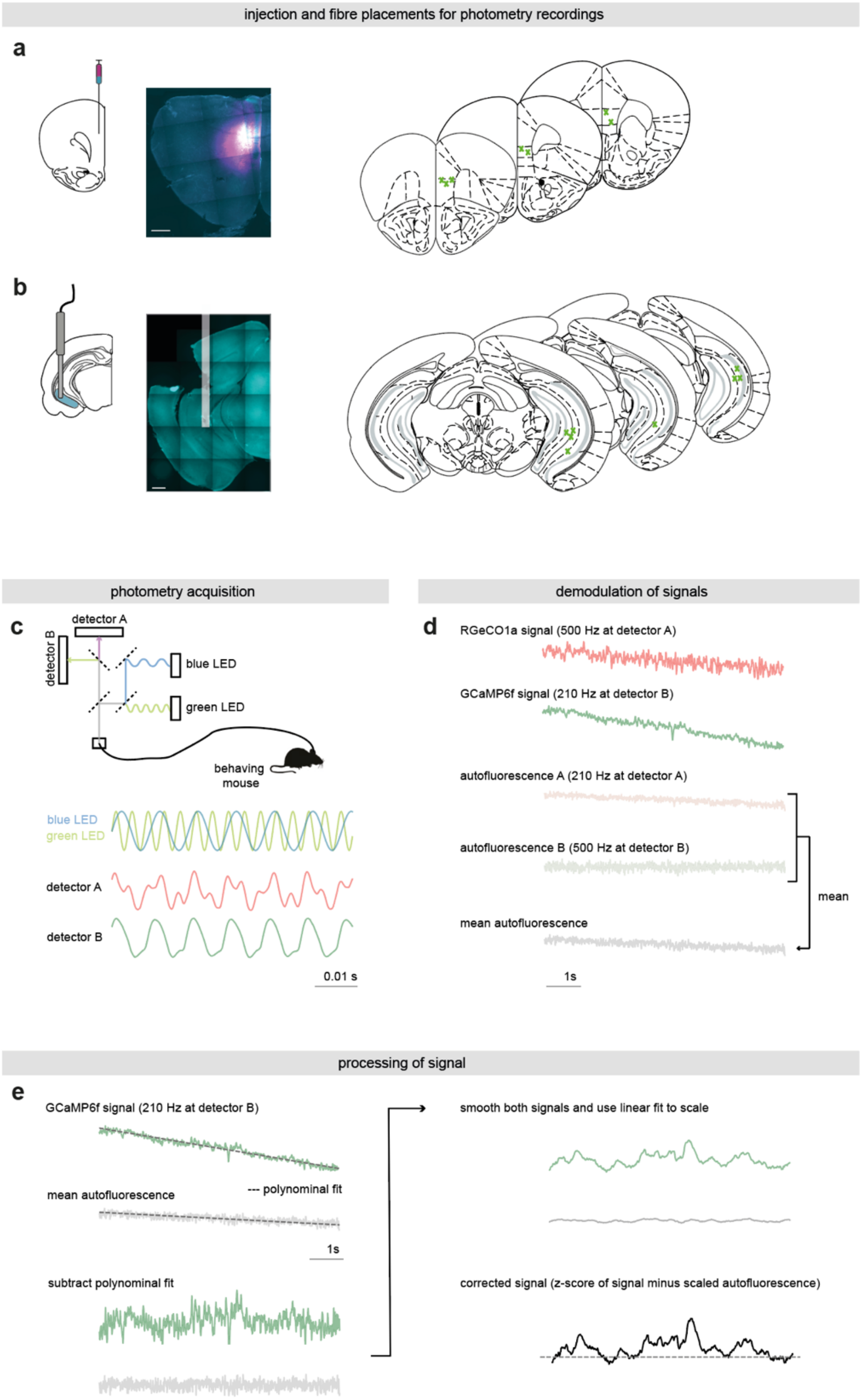

Supp Fig. 9 | Injection sites and protocol for photometry analysis

a) *Left*, schematic and example image of the viral strategy implemented for calcium imaging. Two retrograde calcium sensors were injected in PFC simultaneously in a 50:50 mix - AAVretro creON RGeCO1a and AAVretro creOFF GCaMP6f to achieve expression in the superficial and deep layers of vH, respectively. Image representative of 7 mice. Scale bar = 500*μ*m. *Right,* histology showing the location of the injection sites across all mice.

b) *Left*, schematic and example image of photometry imaging fiber implant in vH. Image representative of 7 mice. Scale bar = 500 *μ*m. *Right*, as in **a)** but for imaging fiber location.

c) Schematic of photometry excitation and acquisition setup. See methods for a detailed explanation.

d) Example demodulation of photometry signal at detectors A and B. The three separated signals (green, red and autofluorescence - the signal from each sensor at the modulation frequency that does not match the sensor) were sampled at 100 kHz, and down sampled to 50 Hz before being exported for further analysis. Mean autofluorescence was taken as the average autofluorescence trace measured at both detectors.

e) Signal processing protocol. A polynomial fit was applied to all signals and subtracted from the raw data to straighten the traces. A least-squares linear fit was then applied to the autofluorescence signal to align it to the green and red signals. Time series data was then calculated for each behavioral session as the z-score of (green or red signal - fitted autofluorescence signal).

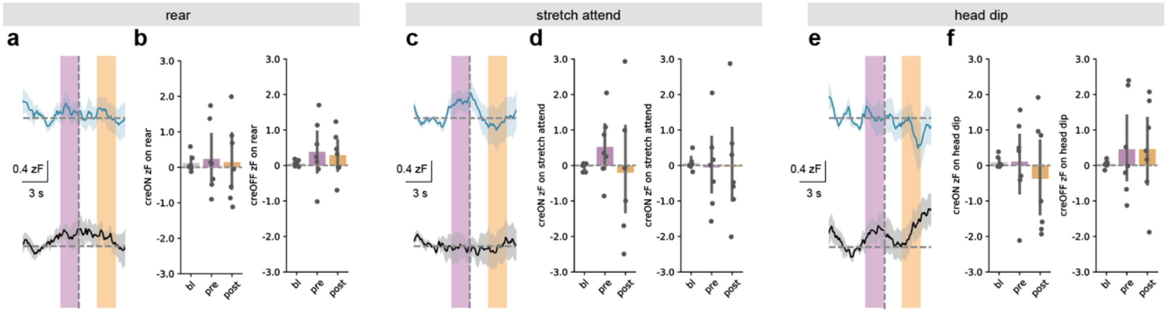

Supp Fig. 10 | Superficial and deep layer vH-PFC activity aligned to distinct behaviors during EPM exploration.

- **a)** Average traces of superficial (blue) and deep (black) layer activity aligned to rearing behavior on the EPM. Note that activity in both is not modulated by rearing bouts.
- **b)** Quantification of changes in activity in creON (superficial, *left*) and creOFF (deep, *right*) cells during the baseline (bl), pre and post-rearing periods (Repeated measures ANOVA, CreON: Main effect of epoch: $F_{(2,12)} = 0.07$, $p = 0.93$; CreOFF: Main effect of epoch: $F_{(2,12)} = 0.94$, $p = 0.42$, $n = 7$ mice).
- **c)** As in **a)** but aligned to stretch attend behavior.
- **d)** As in **b)** but for stretch attend (Repeated measures ANOVA, CreON: Main effect of epoch: $F_{(2,12)} = 1.14$, $p = 0.35$; CreOFF: Main effect of epoch: $F_{(2,12)} = 0.11$, $p = 0.89$, $n = 7$ mice).
- **e)** As in **a)** and **c)** but aligned to head dip behavior.
- **f)** As in **b)** and **d)** but for head dips (Repeated measures ANOVA, CreON: Main effect of epoch: $F_{(2,12)} = 0.10$, $p = 0.90$; CreOFF: Main effect of epoch: $F_{(2,12)} = 0.43$, $p = 0.66$, $n = 7$ mice).

Line plots show mean (line) and SEM (shading and error bars), bar charts show mean (bar) and SEM (error).

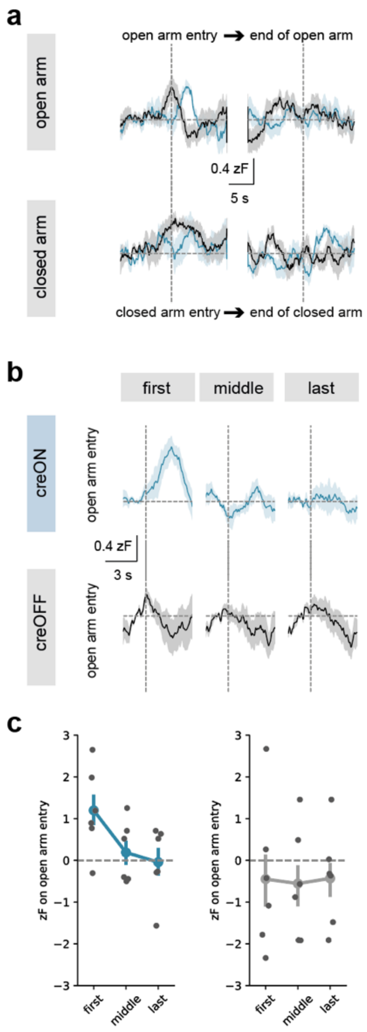

Supp Fig. 11 | Evolution of superficial and deep layer vH-PFC activity during EPM exploration

a) Superficial (blue) and deep (black) layer activity throughout the duration of open (*top*) and closed (*bottom*) arm exploration. Activity in the left panel is aligned to the respective arm entry and the right panel is aligned to the end of the arm before the animal turns to return. Note that activity changes around arm entry but has returned to baseline by the end of the arm.

b) Activity in superficial (blue, creON) and deep (black, creOFF) layers during the first, middle and last entries into the open arms. Note that while the decrease in deep layer activity upon open arm entry is sustained across the session, the transient increase in superficial layer activity is strongest upon the first arm entry and returns to baseline levels towards the last open arm entry.

c) Quantification of normalized calcium signal aligned to the first, middle and last open arm entry for superficial (*left*) and deep (*right*) layer cells (Repeated measures ANOVA, Main effect of epoch, creON: $F_{(2,10)} = 4.66$, $p = 0.04$; creOFF: $F_{(2,10)} = 0.02$, $p = 0.98$, n = 6 mice).

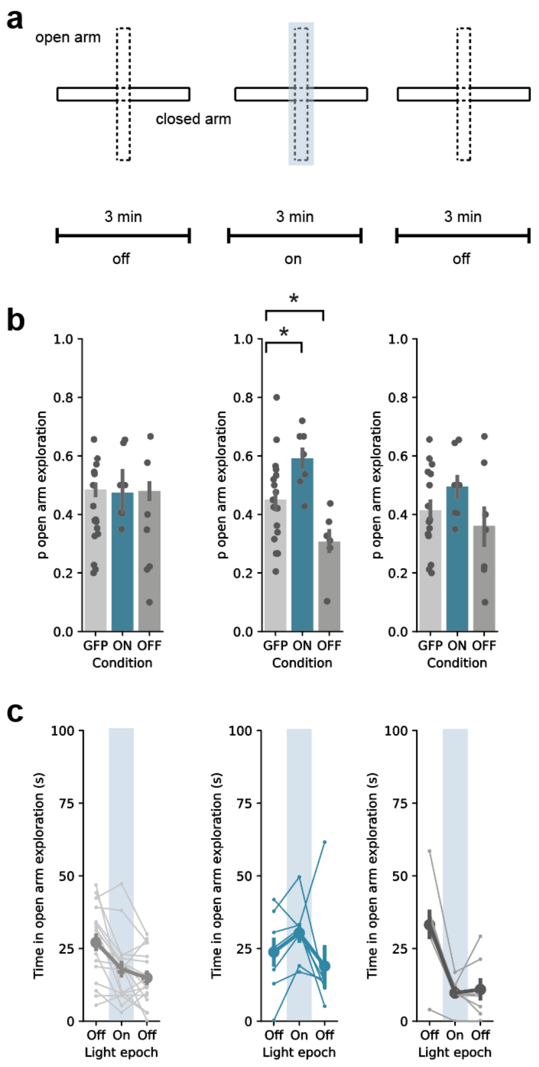

Supp Fig. 12 | Behavioral response to light stimulation during EPM exploration

a) Experimental design: after a 3 min baseline, for a second 3 min epoch 20 Hz light was delivered via the optical fiber when the mouse entered the center point of the maze, and continued until return to the closed arms. Mice then remained in the maze for a third post stimulation 3 min epoch.

b) Proportion of time spent in open arm exploration during the first (*left*), second light-paired (*middle*) and last (*right*) 3 min epochs. Note that behavior is only affected during the second epoch which is paired with light stimulation in the center and open arm portions of the maze (Mixed ANOVA, interaction between epoch and group : $F_{(4,56)} = 2.71$, $p = 0.03$; planned two-sided Welch's paired t-test: Pre: GFP vs creON, $t_{(7)} = 0.13$, [N.S.]$p = 0.90$; GFP vs creOFF, $t_{(10.8)} = 0.12$, [N.S.]$p = 0.91$; Stim: GFP vs creON, $t_{(16.6)} = 2.6$, *$p = 0.04$; GFP vs creOFF, $t_{(11.9)} = 2.4$, *$p = 0.03$; Post: GFP vs creON, $t_{(13.6)} = 1.4$, [N.S.]$p = 0.18$; GFP vs creOFF, $t_{(8.6)} = 0.6$, [N.S.]$p = 0.55$, $n = 17$ (GFP), 7 (creON), 7 (creOFF) mice).

c) Raw and mean traces of time spent in open arm exploration for GFP (grey, *left*), creON (blue, *middle*) and creOFF (grey, *right*) mice. Note that creON (superficial) stimulation leads to an increase and creOFF (deep) stimulation leads to a decrease in open arm exploration relative to controls (GFP).

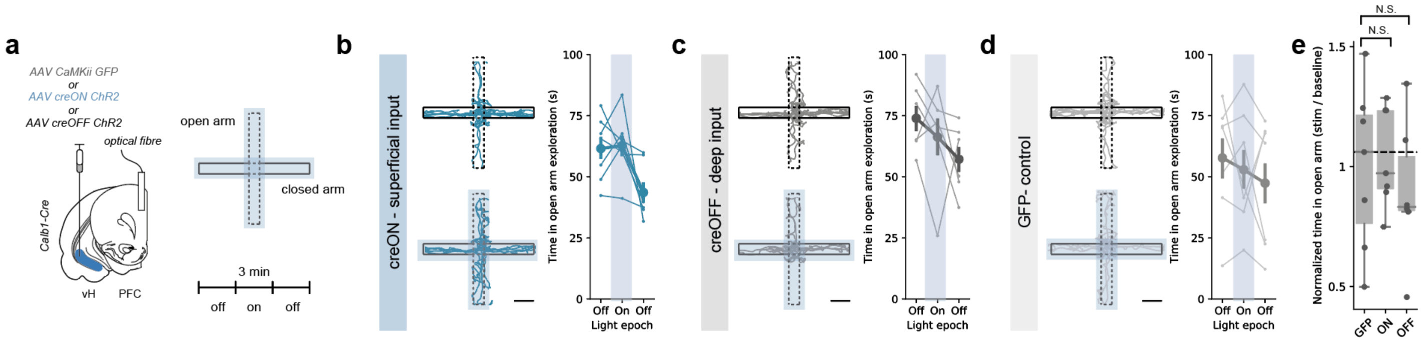

Supp Fig. 13 | Non-location dependent light stimulation does not influence behavior in the EPM

- **a)** *Left,* strategy for *in vivo* optogenetic manipulation of vH axons from each layer in PFC. *Right,* experimental design: after a 3 min baseline, for a second 3 min epoch 20 Hz light was delivered via the optical fiber for the entire 3 min duration. Mice then remained in the maze for a third post stimulation 3 min epoch.
- **b)** Superficial (creON) stimulation in PFC. *Left,* trajectories of an example mouse during baseline (top) and during stimulation (bottom). *Right,* change in open arm exploration due to stimulation. Scale bar = 10 cm.
- **c)** As in **b)** but for stimulation of creOFF (deep) input to PFC.
- **d)** As in **b)** and **c)** but for stimulation in GFP control animals.
- **e)** Summary of the effect of activation on open arm exploration. Although the trend of decreased exploration following creOFF stimulation is consistent with location-dependent stimulation shown in **Figure 6**, constant stimulation did not significantly influence open arm exploration for either creON (two-sided Mann Whitney, U = 22, p = 0.80, n = 7 (GFP), 7 (creON) mice) or creOFF (two-sided Mann Whitney, U = 26, p = 0.52, n = 7 (GFP), 6 (creOFF) mice)conditions relative to controls. Dotted line shows median exploration of GFP controls for comparison. See supplementary statistics table for further information.

Box plots show median, 75th (box) and 95th (whiskers) percentile. Line plots show mean (line) and SEM (shading and error bars).

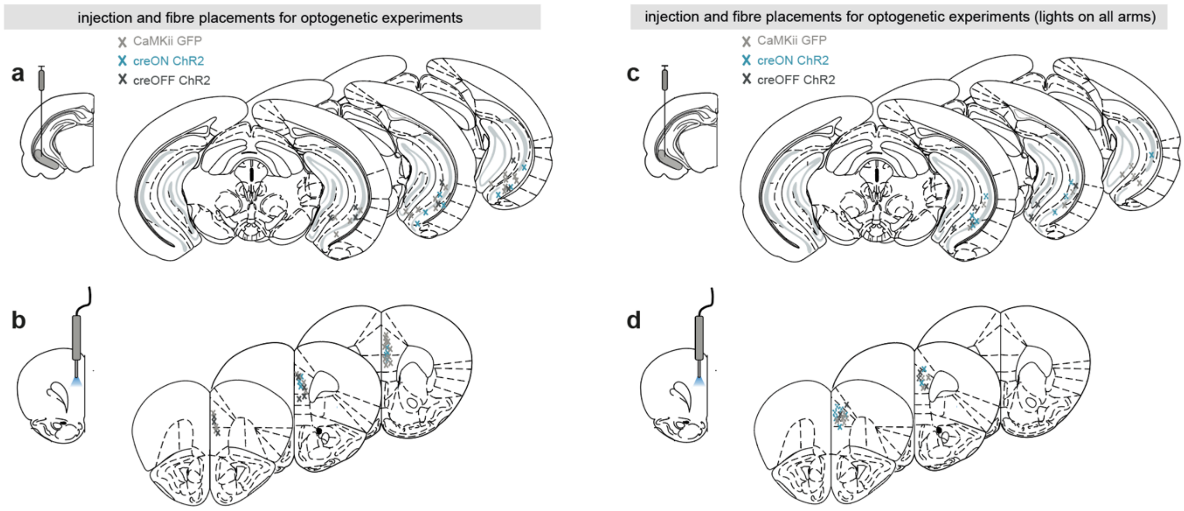

Supp Fig. 14 | Injection site and fiber placements for *in-vivo* optogenetics

- **a)** *Left*, schematic of injections of creON ChR2, creOFF ChR2 and GFP into vH. *Right*, histology showing the location of the injection sites across all mice.
- **b)** *Left*, schematic of optogenetic fiber placement in PFC. *Right*, histology showing the location of the fiber implant placements across all mice.
- **c)** As in **a)** but for mice used in non-location dependent stimulation during EPM exploration.
- **d)** As in **b)** but for mice used in non-location dependent stimulation during EPM exploration.

SUPPLEMENTARY STATISTICAL TEST SUMMARY FOR MAIN FIGURES

FIGURE	DESCRIPTORS	n	TEST USED	STATISTIC	P-VALUE
**2c**	Apical dendrite length with increasing distance from alveus (μm)	Sup: 7<br>Deep: 6<br><br>(neurons)	Repeated measures ANOVA<br>Main effect of group:	F(35,385) = 1.549	p = 0.027
	Basal dendrite length with increasing distance from alveus (μm)	Sup: 7<br>Deep: 6<br><br>(neurons)	Repeated measures ANOVA w/ Greenhouse-Geisser correction<br>Main effect of group:	F(33,367) = 1.701	p = 0.179
**2d**	Longest dendrite (μm)	Sup: 7<br>Deep: 6<br><br>(neurons)	Mann Whitney	U = 35.5	p = 0.045
	Distance of bifurcation (μm)	Sup: 7<br>Deep: 6<br><br>(neurons)	Mann Whitney	U = 38.5	p = 0.015
**2g**	# action potentials with increasing current step (pA)	Sup: 22<br>Deep: 26<br><br>(neurons)	Repeated measures ANOVA w/ Greenhouse-Geisser correction<br>Interaction between group and current step:	F(1.4, 44.5) = 4.3	p = 0.032
**2h**	% bursting neurons	Sup: 22<br>Deep: 26<br><br>(neurons)	Fishers Exact	Odds ratio = 12.5	p = 0.009
	Sag amplitude (mV)	Sup: 22<br>Deep: 26<br><br>(neurons)	Mann Whitney	U = 39.5	p = 0.002
**3a**	CA3 sup : deep amp at 5 ms (mV)	10 (neurons)	Wilcoxon Paired	W = 27	p = 1.0
**3b**	ENT sup : deep amp at 5 ms (mV)	9 (neurons)	Wilcoxon Paired	W = 44	p = 0.007
**3c**	ATh sup : deep amp at 5 ms (mV)	7 (neurons)	Wilcoxon Paired	W = 12	p = 0.034
**3d**	DBB sup : deep amp at 5 ms (mV)	15 (neurons)	Wilcoxon Paired	W = 24	p = 0.041
**3e**	PV PFC+ sup:deep charge at 5 ms (C)	17 (neurons)	Wilcoxon Paired	W = 57	p = 0.38
**3f**	PV PFC- sup:deep charge at 5 ms (C)	11 (neurons)	Wilcoxon Paired	W = 10	p = 0.042
**3i**	% connected	Sup: 19<br>Deep: 18<br><br>(neurons)	Fishers Exact	Odds ratio = 0.95	p = 1.0
	Amplitude of connection if present (pA)	Sup: 5<br>Deep: 5<br><br>(neurons)	Mann Whitney	U = 8	p = 0.42
**4c**	creON vs creOFF<br>Axon distribution across DV<br><br><br><br><br>Axon distribution across ML	4 (injections)	Repeated measures ANOVA<br>Main effect of group:<br>Main effect of DV:<br>Interaction:<br><br>Repeated measures ANOVA<br>Main effect of group:	F(1,6) = 0.31<br>F(20,120) = 9.84<br>F(20,120) = 0.48<br><br><br>F(1,6) = 0.10	p = 0.599<br>p = 0.00<br>p = 0.97<br><br><br>p = 0.77

	Axon distribution across AP		Main effect of ML:<br>Interaction:<br><br>Repeated measures ANOVA<br>Main effect of group:<br>Main effect of AP:<br>Interaction:	F(11,66) = 34.2<br>F(11,66) = 0.82<br><br><br>F(1,6) = 0.03<br>F(10,60) = 27.0<br>F(10,60) = 1.44	p = 0.00<br>p = 0.62<br><br><br>p = 0.59<br>p = 0.00<br>p = 0.19
**4f**	CTX *p* superficial	CaMKii: 4<br>VGAT: 4<br>(injections)	Mann Whitney	U = 4	p = 0.3
	Rabies *p* superficial	CaMKii: 4<br>VGAT: 4<br>(injections)	Mann Whitney	U = 16	p = 0.03
**4i**	creON vs creOFF<br>-70 mV charge at 5 ms (C)	creON: 10<br>creOFF: 8<br><br>(neurons)	Mann Whitney	U = 50	p = 0.41
	creON vs creOFF<br>0 mV charge at 5 ms (C)	creON: 10<br>creOFF: 8<br><br>(neurons)	Mann Whitney	U = 91	p = 0.01
	creON vs creOFF<br>E : I ratio at 5 ms	creON: 10<br>creOFF: 8<br><br>(neurons)	Mann Whitney	U = 10	p = 0.006
**4l**	creON FS-: FS+ charge at 5 ms (C)	10 (neurons)	Wilcoxon Paired	W = 35	p = 0.49
**4m**	creOFF FS-: FS+ charge at 5 ms (C)	15 (neurons)	Wilcoxon Paired	W = 111	p = 0.02
**5f**	creON<br>zF bl, pre and post open arm entry<br><br><br>bl vs pre<br><br>bl vs post	7 (mice)	Repeated measures ANOVA<br>Main effect of epoch:<br><br>Welch Paired ttest<br><br>Welch Paired ttest	 F(2, 12) = 4.52<br><br>t(6) = 0.3<br><br>t(6) = 4.4	 p = 0.034<br><br>p = 0.77<br><br>p = 0.004
**5f**	creOFF<br>zF bl, pre and post open arm entry<br><br><br>bl vs pre<br><br>bl vs post	7 (mice)	Repeated measures ANOVA<br>Main effect of epoch:<br><br>Welch Paired ttest<br><br>Welch Paired ttest	 F(2, 12) = 13.4<br><br>t(6) = 2.5<br><br>t(6) = 3.4	 p = 0.001<br><br>p = 0.04<br><br>p = 0.02
**5h**	creON<br>zF bl, pre and post closed arm entry<br><br><br>bl vs pre<br><br>bl vs post	7 (mice)	Repeated measures ANOVA<br>Main effect of epoch:<br><br>Welch Paired ttest<br><br>Welch Paired ttest	 F(2, 12) = 0.11<br><br>t(6) = 0.8<br><br>t(6) = 0.06	 p = 0.90<br><br>p = 0.43<br><br>p = 0.95
**5h**	creOFF<br>zF bl, pre and post closed arm entry<br><br><br>bl vs pre<br><br>bl vs post	7 (mice)	Repeated measures ANOVA<br>Main effect of epoch:<br><br>Welch Paired ttest<br><br>Welch Paired ttest	 F(2, 12) = 4.12<br><br>t(6) = 0.8<br><br>t(6) = 4.2	 p = 0.043<br><br>p = 0.44<br><br>p = 0.01
**5j**	creON – creOFF<br>zF open vs closed arm<br><br>pre arm entry<br><br>post arm entry	7 (mice)	 <br><br>Welch Paired ttest<br><br>Welch Paired ttest	 <br><br>t(6) = 0.6<br><br>t(6) = 3.1	 <br><br>p = 0.59<br><br>p = 0.02
**6e**	Time exploring open arm (s)	GFP: 17<br>creON: 7<br>creOFF: 7	Mixed ANOVA<br>Main effect of group:	 F(2, 28) = 0.81<br>F(2, 56) = 15.9	 p = 0.45<br>p = 0.00

		(mice)	Main effect of epoch:<br>Interaction:	F(4, 56) = 4.78	p = 0.002
	Pre GFP vs creON<br>Pre GFP vs creOFF<br><br>Stim GFP vs creON<br>Stim GFP vs creOFF<br><br>Post GFP vs creON<br>Post GFP vs creOFF		Welch ttest<br>Welch ttest<br><br>Welch ttest<br>Welch ttest<br><br>Welch ttest<br>Welch ttest	t(10) = 0.5<br>t(9.3) = 0.4<br><br>t(11.9) = 2.5<br>t(21.7) = 2.4<br><br>t(7.3) = 0.5<br>t(10.2) = 0.9	p = 0.61<br>p = 0.39<br><br>p = 0.03<br>p = 0.04<br><br>p = 0.61<br>p = 0.40
**6f**	Normalised open arm exploration	GFP: 17<br>creON: 7<br>(mice)	Mann Whitney	U = 12	p = 0.002
	Normalised open arm exploration	GFP: 17<br>creOFF: 7<br>(mice)	Mann Whitney	U = 100	p = 0.008

SUPPLEMENTARY STATISTICAL TEST SUMMARY FOR SUPPLEMENTARY FIGURES

FIGURE	DESCRIPTORS	n	TEST USED	STATISTIC	P-VALUE
**S2b**	Proportion of colocalized cells PFC:NAc vs PFC:LH	NAc: 13<br>LH: 21<br><br>(slices)	Welch ttest	t(13.6) = 3.5	p = 0.004
**S2c**	Proportion colocalized cells / layer<br><br>PFC:NAc<br><br>PFC:LH	 <br><br>11<br><br>21<br><br>(slices)	 <br><br>Welch ttest<br><br>Welch ttest	 <br><br>t(20) = 5.6<br><br>t(40) = 6.7	 <br><br>p = 0.00002<br><br>$p = 5.3x10^{-8}$
**S3e**	Proportion superficial Cb+ vs Cb-	4<br><br>(injections)	Welch ttest	t(6) = 7.2	p = 0.0004
**S4c**	Distance from lateral alveus (μm)	Sup: 7<br>Deep: 6<br><br>(neurons)	Mann Whitney	U = 0	p = 0.003
	Distance from medial avleus (μm)	Sup: 7<br>Deep: 6<br><br>(neurons)	Mann Whitney	U = 39	p = 0.012
**S4e**	Longest dendrite (μm)	Sup: 7<br>Deep: 6<br><br>(neurons)	Mann Whitney	U = 35.5	p = 0.045
	Distance of bifurcation (μm)	Sup: 7<br>Deep: 6<br><br>(neurons)	Mann Whitney	U = 38.5	p = 0.015
**S4g**	# action potentials with increasing current step (pA)	Sup: 22<br>Deep: 26<br><br>(neurons)	Repeated measures ANOVA w/ Greenhouse-Geisser correction<br>Interaction between group and current step:	F(1.4, 44.5) = 4.3	p = 0.032
**S4h**	% bursting neurons	Sup: 22<br>Deep: 26<br><br>(neurons)	Fishers Exact	Odds ratio = 12.5	p = 0.009
**S4i**	Threshold (mV)	Sup: 22<br>Deep: 26<br><br>(neurons)	Mann Whitney	U = 219	p = 0.169
	AP amplitude (mV)	Sup: 22<br>Deep: 26<br><br>(neurons)	Mann Whitney	U = 423	p = 0.005
	AP width (ms)	Sup: 22<br>Deep: 26<br><br>(neurons)	Mann Whitney	U = 207	p = 0.104
	AHP Depth (mV)	Sup: 22<br>Deep: 26<br><br>(neurons)	Mann Whitney	U = 143	p = 0.003
**S4j**	Input resistance (MΩ)	Sup: 22<br>Deep: 26<br><br>(neurons)	Mann Whitney	U = 393	p = 0.028
	Sag amplitude (mV)	Sup: 22<br>Deep: 26<br><br>(neurons)	Mann Whitney	U = 39.5	p = 0.002

**S8e**	SC sup : deep amp at 0.5 ms (mV)	10 (neurons)	Wilcoxon Paired	W = 23	p = 0.646
**S8f**	TA sup : deep amp at 0.5 ms (mV)	14 (neurons)	Wilcoxon Paired	W = 19	p = 0.035
**S10b**	creON<br>zF bl, pre and post rear<br><br>creOFF<br>zF bl, pre and post rear	7 (mice)	Repeated measures ANOVA<br>Main effect of epoch:<br><br>Repeated measures ANOVA<br>Main effect of epoch:	F(2, 12) = 0.07<br><br>F(2, 12) = 0.94	p = 0.93<br><br>p = 0.42
**S10d**	creON<br>zF bl, pre and post stretch attend<br><br>creOFF<br>zF bl, pre and post stretch attend	7 (mice)	Repeated measures ANOVA<br>Main effect of epoch:<br><br>Repeated measures ANOVA<br>Main effect of epoch:	F(2, 12) = 1.14<br><br>F(2, 12) = 0.11	p = 0.35<br><br>p = 0.89
**S10f**	creON<br>zF bl, pre and post head dip<br><br>creOFF<br>zF bl, pre and post head dip	7 (mice)	Repeated measures ANOVA<br>Main effect of epoch:<br><br>Repeated measures ANOVA<br>Main effect of epoch:	F(2, 12) = 0.10<br><br>F(2, 12) = 0.43	p = 0.90<br><br>p = 0.66
**S11c**	creON first, middle last arm entry<br><br>creOFF first, middle, last arm entry	6 (mice)	Repeated measures ANOVA<br>Main effect of epoch:<br><br>Repeated measures ANOVA<br>Main effect of epoch:	F(2,10) = 4.66<br><br>F(2,10) = 0.02	p = 0.04<br><br>p = 0.98
**S12b**	p open arm entry<br><br><br><br>Pre GFP vs creON<br>Pre GFP vs creOFF<br><br>Stim GFP vs creON<br>Stim GFP vs creOFF<br><br>Post GFP vs creON<br>Post GFP vs creOFF	GFP: 17<br>creON: 7<br>creOFF: 7<br>(mice)	Mixed ANOVA<br>Main effect of group:<br>Main effect of epoch:<br>Interaction:<br><br>Welch ttest<br>Welch ttest<br><br>Welch ttest<br>Welch ttest<br><br>Welch ttest<br>Welch ttest	 F(2, 28) = 2.11<br>F(2, 56) = 1.15<br>F(4, 56) = 2.71<br><br>t(7.0) = 0.13<br>t(10.8) = 0.12<br><br>t(16.6) = 2.6<br>t(11.9) = 2.4<br><br>t(13.6) = 1.4<br>t(8.6) = 0.6	 p = 0.14<br>p = 0.32<br>p = 0.03<br><br>p = 0.90<br>p = 0.91<br><br>p = 0.04<br>p = 0.03<br><br>p = 0.18<br>p = 0.55
**S13**	Time spent in open arm (s)	GFP: 7<br>creON: 7<br>creOFF: 6<br>(mice)	Mixed ANOVA<br>Main effect of group:<br>Main effect of epoch<br>Interaction:	 F(2, 17) = 1.36<br>F(2, 34) = 6.00<br>F(4, 34) = 0.50	 p = 0.28<br>p = 0.01<br>p = 0.74
**S13**	Normalized open arm exploration	GFP: 7<br>creON: 7<br>(mice)	Mann Whitney	U = 22	p = 0.80
	Normalized open arm exploration	GFP: 7<br>creOFF: 6<br>(mice)	Mann Whitney	U = 26	p = 0.52
