# Supplementary material for: Two opposing hippocampus to prefrontal cortex pathways for the control of approach and avoidance behaviour
Source: Nat Commun. 2022 Jan 17;13:339. doi: 10.1038/s41467-022-27977-7 (PMC8763938; doi:10.1038/s41467-022-27977-7)
Supplement: Supplementary file 3 — Reporting Summary [file 41467_2022_27977_MOESM3_ESM.pdf]

natureresearch

Corresponding author(s): Andrew F MacAskill

Last updated by author(s): Dec 2, 2021

Reporting Summary

Nature Research wishes to improve the reproducibility of the work that we publish. This form provides structure for consistency and transparency in reporting. For further information on Nature Research policies, see Authors & Referees and the Editorial Policy Checklist.

Statistics

For all statistical analyses, confirm that the following items are present in the figure legend, table legend, main text, or Methods section.

n/a	Confirmed	
☐	☒	The exact sample size (*n*) for each experimental group/condition, given as a discrete number and unit of measurement
☐	☒	A statement on whether measurements were taken from distinct samples or whether the same sample was measured repeatedly
☐	☒	The statistical test(s) used AND whether they are one- or two-sided<br>*Only common tests should be described solely by name; describe more complex techniques in the Methods section.*
☐	☒	A description of all covariates tested
☐	☒	A description of any assumptions or corrections, such as tests of normality and adjustment for multiple comparisons
☐	☒	A full description of the statistical parameters including central tendency (e.g. means) or other basic estimates (e.g. regression coefficient) AND variation (e.g. standard deviation) or associated estimates of uncertainty (e.g. confidence intervals)
☐	☒	For null hypothesis testing, the test statistic (e.g. *F*, *t*, *r*) with confidence intervals, effect sizes, degrees of freedom and *P* value noted<br>*Give P values as exact values whenever suitable.*
☒	☐	For Bayesian analysis, information on the choice of priors and Markov chain Monte Carlo settings
☒	☐	For hierarchical and complex designs, identification of the appropriate level for tests and full reporting of outcomes
☒	☐	Estimates of effect sizes (e.g. Cohen's *d*, Pearson's *r*), indicating how they were calculated

Our web collection on statistics for biologists contains articles on many of the points above.

Software and code

Policy information about availability of computer code

Data collection	Electrophysiology recordings were acquired using WinWCP V 5.1.8 (University of Strathclyde), and two-photon imaging was carried out using SciScan (Scientifica). Histology was acquired and visualized using Zen software (Zeiss). A .vi using standard functions in Labview v15 (National Instruments) was used to for photometry data acquisition. This code is available at https://github.com/macaskill-lab
Data analysis	Data analysis was performed in Python 3.6 as described in the methods. We used standard python packages: numpy (v1.18.5), pandas (v1.0.5), scipy (v1.5.0), matplotlib (v3.2.2), seaborn (v0.10.1), as well as scikit.learn (v0.23.1), neo (v0.9.0), and efel (v3.1.50). 2-photon imaging was analyzed using Volume Integration and Alignment System (VIAS) v2.4 and Neuron Studio v 0.9.92 (both CNIC, Mount Sinai). Image analysis was carried out using FIJI (ImageJ version 2.0.0-rc-68/1.52i) and associated plugins. Behavior was analyzed using EthoVision XT (Noldus), and deeplabcut (v2.0) (Mathis et al. 2018 (ref87)). Whole brain anatomy was analysed using the Wholebrain package (v0.1.35) in R (v3.5.2). Statistical tests were performed using pingouin (v0.4.0) in python.

For manuscripts utilizing custom algorithms or software that are central to the research but not yet described in published literature, software must be made available to editors/reviewers. We strongly encourage code deposition in a community repository (e.g. GitHub). See the Nature Research guidelines for submitting code & software for further information.

Data

Policy information about availability of data

All manuscripts must include a data availability statement. This statement should provide the following information, where applicable:

- Accession codes, unique identifiers, or web links for publicly available datasets
- A list of figures that have associated raw data
- A description of any restrictions on data availability

Source data are provided for Figure 1 - 6, and are available at https://github.com/macaskill-lab. Due to volume, raw data are available from the corresponding author (a.macaskill@ucl.ac.uk) upon reasonable request. Data from the Allen reference atlas (https://mouse.brain-map.org/static/atlas) were used in this study.

Field-specific reporting

Please select the one below that is the best fit for your research. If you are not sure, read the appropriate sections before making your selection.

☒ Life sciences ☐ Behavioural & social sciences ☐ Ecological, evolutionary & environmental sciences

For a reference copy of the document with all sections, see nature.com/documents/nr-reporting-summary-flat.pdf

Life sciences study design

All studies must disclose on these points even when the disclosure is negative.

Sample size	No statistical test was run to determine sample size a priori. The sample sizes chosen are similar to those used in previous publications. For electrophysiology this based on previous, similar work (MacAskill et al, 2014 (ref 40)). For optogenetics and photometry, sample sizes were based on previous literature (e.g. Felix-Ortiz et al, 2013, Pi et al, 2020 (ref 7), Padilla-Coreano et al, 2019 (ref 11)).
Data exclusions	Animals were excluded if either injection or fiber placement was not accurate. To avoid ceiling effects in optogenetic behavioral experiments, mice were excluded if they failed to enter either open arm in the first three minutes of testing. This exclusion criterion was pre-established before the start of the experiment (see ref 88).
Replication	Reproducibility was ensured by sampling from multiple biological replicates, i.e., from multiple mice. No results are included that were not replicated across at least 3 experiments (mice).
Randomization	Animals were randomly assigned to a virus cohort (e.g. ChR2 versus GFP). Littermates were split evenly into virus cohorts to avoid cage-specific effects. For electrophysiology experiments the order of recording sequential superficial/deep or pyramidal cell/interneuron pairs was alternated to eliminate any potential bias.
Blinding	For behavioral experiments, the experimenter was blinded to virus assignment when the experiment was performed and analysed. Due to the presence of injection sites in in vitro slices, for non behavioral experiments the experimenter was not blinded at either acquisition or analysis.

Reporting for specific materials, systems and methods

We require information from authors about some types of materials, experimental systems and methods used in many studies. Here, indicate whether each material, system or method listed is relevant to your study. If you are not sure if a list item applies to your research, read the appropriate section before selecting a response.

Materials & experimental systems

n/a	Involved in the study	
☒	☐	Antibodies
☒	☐	Eukaryotic cell lines
☒	☐	Palaeontology
☐	☒	Animals and other organisms
☒	☐	Human research participants
☒	☐	Clinical data

Methods

n/a	Involved in the study	
☒	☐	ChIP-seq
☒	☐	Flow cytometry
☒	☐	MRI-based neuroimaging

Animals and other organisms

Policy information about studies involving animals; ARRIVE guidelines recommended for reporting animal research

Laboratory animals	Male and female wild-type C57 / bl6J mice (6-10 weeks) provided by Charles River were used except where noted. To target inhibitory neurons we used the Slc32a1(VGAT)-IRES-Cre (#016962) knock-in line. To target Calbindin 1 expressing neurons used the Calb1-IRES-Cre (#028532) knock-in line. To target parvalbumin positive interneurons we used the PV-IRES-Cre (#008069) knock in line. All were obtained from Jackson laboratories and bred in-house. Mice were housed in cages of 2 - 4 and kept in a humidity- (45 - 60 %) and temperature- (20-24 celsius) controlled environment under a 12 h light/dark cycle (lights on 7 am to 7 pm) with ad-libitum access to food and water.
Wild animals	No wild animals were used in this study.
Field-collected samples	No Field collected samples were used in this study.
Ethics oversight	All experiments were approved by the U.K. Home Office as defined by the Animals (Scientific Procedures) Act, and by the University College London Animal Welfare Ethical Review Body.

Note that full information on the approval of the study protocol must also be provided in the manuscript.
